# Typhoid Fever: Treatment in the Army

**Published:** 1916-04-15

**Authors:** 


### TYPHOID FEVER.

### TREATMENT IN THE ARMY.

On previous occasions the distinctions between the three fevers of the enteric group—typhoid fever, paratyphoid A fever, and paratyphoid B fever—have been explained; and the *rationale* of the methods of preventive inoculation against one or all three of these diseases has been discussed. This prophylactic use of vaccines against infections by the "enteric group" organisms is not the only method of employing them, however; there is also a method of using vaccine in the treatment of the fully developed diseases. The present writer's personal experience of this usage is confined to some three or four cases; and upon so slender a foundation it would be extremely unsafe to build any superstructure of dogmatism. Quite recently, however, a paper embodying by far the most extensive experience yet analysed of vaccine treatment for typhoid fever has been published (Captain T. H. Whittington. R.A.M.C.; *Lancet*, April 8, 1916, p. 759), and the number of cases is sufficiently large for results to be stated with confidence and authority.

### Prevention and Cure.

The preventive treatment of the enteric fevers consists of the injection under the skin of a small quantity of fluid containing a certain number of millions of dead bacilli, grown in the laboratory on suitable food and killed either by heat or by clinical substances which are poisonous to them. If the typhoid bacillus is used, the man is protected to a high degree, but not absolutely, against typhoid fever; if a paratyphoid bacillus, against the corresponding fever; and if a suitable mixture of typhoid and paratyphoid organisms is used, then he is protected against all three fevers. This result is believed to be due to the response of the body to the presence of enteric bacilli and their products, whereby substances are formed which are antagonistic to the bacilli. These anti-bodies remain present in the body for many months or a few years, gradually diminishing in amount; and while they are present their host is immune, or partially immune, to fresh invasions of live bacilli: in other words, he is not so likely to contract enteric fever when exposed to its infection. The theory of the business, it will be seen, is simple and comprehensible, and it is based upon experimental evidence which, though not infallible, is so strong as to carry conviction to all who have made themselves acquainted with it.

The theory of the vaccine treatment of enteric fever itself, as opposed to the preventive inoculation of healthy individuals, is much less simple and satisfactory. To the plain man it would seem that when a patient is coping to the best of his body's abilities with an invasion of millions of live enteric bacilli, it is not very likely to help him if many millions more of dead bacilli (with their products) are introduced into the fray. There are, however, complicated arguments forthcoming from the bacteriologist to explain why vaccine treatment should, in spite of its apparent lack of logic, be of great benefit. These arguments lack the simplicity of those on which prophylactic treatment is based; nor is the experimental evidence nearly so positive or so pregnant with conviction. The ordinary clinician, of course, demands practical results before he will accept any of these methods. In the case of preventive inoculation such results have long been supplied; they constitute the very highest possible testimony to the process, and have unquestionably saved thousands—nay, hundreds of thousands—of our soldiers' lives during the campaign in Europe. In the case of curative vaccine treatment the practical results have not hitherto been available in sufficient numbers to justify positive statements; though the opinion of many of those who have tried it has been distinctly unfavourable. Now, thanks to Captain Whittington, a trial on a sufficiently large scale has taken place, and the results have been analysed with care and precision. In order to exclude the influence of protective inoculation, his statistics deal chiefly with the uninoculated, a limitation the more easy to establish since the uninoculated provided him with the bulk of his clinic. He took two hundred uninoculated patients, all suffering from true typhoid fever, and treated alternate ones with vaccine, thus obtaining one hundred vaccine-treated cases to compare with the same number treated on ordinary lines.

### A Summary of Results.

In a hundred and fifteen vaccine-treated patients, fifteen of whom had been inoculated and the others not, he found twenty-nine in whom the vaccine appeared to have a definite good influence; the great majority of these were mild cases, in which a good result was to be expected in any event. In severe cases no good effect could be traced, and a suspicion was entertained that in some of them the vaccine did harm. Vaccine, says Captain Whittington, neither shortens the fever nor reduces the number of complications in even that class of case which is likely to do well. Vaccine is "disappointing" in just those cases (the severe ones) in which the physician most requires help. Vaccine possibly increases the tendency to intestinal hæmorrhage. Such are the conclusions of an observer who has been exceptionally well placed for noting the results of vaccine treatment. Since stock vaccine cannot be recommended as a routine part of the treatment of typhoid fever, it remains to be seen whether autogenous vaccines would do any better. Inasmuch as the great success of prophylactic tratment has been gained necessarily with stock vaccines, it may be doubted whether there is any great likelihood of autogenous proving superior to stock vaccines in typhoid fever. Nor are the conditions of active service favourable to the preparation and employment of autogenous vaccines.

